# Supplementary material for: Unified methods in collecting, preserving, and archiving coral bleaching and restoration specimens to increase sample utility and interdisciplinary collaboration
Source: PeerJ. 2022 Nov 2;10:e14176. doi: 10.7717/peerj.14176 (PMC9636870; doi:10.7717/peerj.14176)
Supplement: Supplemental Information 1 — Unified methods in collecting, preserving, and archiving coral bleaching and restoration specimens to increase sample utility and interdisciplinary collaboration [file peerj-10-14176-s001.docx]

**SUPPLEMENTAL MATERIALS**

**Unified methods in collecting, preserving, and archiving coral bleaching and restoration specimens to increase sample utility and interdisciplinary collaboration**

**Authors**: Rebecca Vega Thurber^1*^, Emily R Schmeltzer^1^, Andréa G Grottoli^2^,  Robert van Woesik^3^, Robert Toonen^4^, Mark E Warner^5^, Kerri L Dobson^2^, Rowan H McLachlan^1,2^, Katie L Barott^6^, Daniel J Barshis^7^, Justin H Baumann^8^, Leila Chapron^2^, David J Combosch^9^, Adrienne MS Correa^10^, Thomas M DeCarlo^11^, Mary Hagedorn^12^,  Laetitia Hedouin^13^, Kenneth D Hoadley^14^, Thomas Felis^15^, Christine Ferrier-Pages^16^, Carly D Kenkel^17^, Ilsa B Kuffner^18^, Jennifer L Matthews^19^, Mónica Medina^20^, Christopher P Meyer^21^, Corinna Oster^15^, James T Price^2^, Hollie M Putnam^22^, Yvonne Sawall^23^

**Affiliations**

1. Department of Microbiology, Oregon State University, Corvallis, OR, USA 97331
2. School of Earth Sciences, The Ohio State University, Columbus, OH 43210, USA
3. Institute for Global Ecology, Florida Institute of Technology, Melbourne, Fl 32901, USA
4. Hawai’i Institute of Marine Biology, SOEST, University of Hawai’i at Mānoa, PO Box 1346, Kāne’ohe, HI 96744
5. School of Marine Science and Policy, University of Delaware, Lewes, DE, 19958, USA
6. Department of Biology, University of Pennsylvania, Philadelphia, PA USA 19104
7. Department of Biological Sciences, Old Dominion University, Norfolk, VA 23529
8. Biology Department, Bowdoin College, Brunswick, ME, 04011, USA
9. Marine Laboratory, University of Guam, 303 University Drive, Mangilao, Guam
10. BioSciences Department, Rice University, Houston, TX 77005
11. College of Natural and Computational Sciences, Hawai’i Pacific University, Honolulu, HI 96813
12. Smithsonian Conservation Biology Institute and Hawai’i Institute of Marine Biology, Kāne’ohe, HI 96744
13. Chargée de Recherches CNRS Centre de Recherches Insulaires et Observatoire de l’Environnement CRIOBE USR 3278BP1013, 98729, Papetō’ai, Mo’orea, Polynesie Française
14. Department of Biological Sciences, University of Alabama, Tuscaloosa, AL, 35487, USA
15. MARUM – Center for Marine Environmental Sciences, University of Bremen, 28359 Bremen, Germany
16. Centre Scientifique de Monaco, 8 Quai Antoine 1°, MC98000 Monaco
17. Department of Biological Sciences, University of Southern California, 3616 Trousdale Parkway, Los Angeles, CA 90089-0371
18. U.S. Geological Survey, St. Petersburg, FL 33701, USA
19. Climate Change Cluster, University of Technology Sydney, Ultimo 2007, NSW, Australia
20. Department of Biology, Pennsylvania State University, University Park, PA 16802, USA
21. Department of Invertebrate Zoology, National Museum of Natural History, Smithsonian Institution, 10th & Constitution Aves. NW, Washington DC 20560, USA
22. Department of Biological Sciences, University of Rhode Island, Kingston, RI 02881, USA
23. Bermuda Institute of Ocean Sciences (BIOS), 17 Biological Station, St. George’s GE01, Bermuda

Corresponding Author* Rebecca Vega Thurber, [rvegathurber@gmail.com](mailto:rvegathurber@gmail.com)

**Contributions and Acknowledgements:**

This workshop was funded by the National Science Foundation Division of Biological Oceanography #1838667 to AGG, RT, RvW, MEW, and RVT. The manuscript concept was developed by RVT, AGG, RT, RvW, MEW, ERS, KLD, and RHM. All authors participated in the Coral Bleaching Research Coordination Network virtual workshop in July 2020 where the content of this manuscript was developed, and all contributed to writing and revising of the manuscript. RVT was the workshop leader, coordinated all writing efforts, compiled all components of the manuscript, and incorporated all revisions with the help of ERS and RHM. Any use of trade, firm, or product names is for descriptive purposes only and does not imply endorsement by the U.S. Government.

**Discipline-Based Methods Appendices:**

**Omics-Based Methods**

DNA-Based Work

*Sample Collection*: DNA samples should be collected using aseptic techniques to maximize the number of analyses that can be completed without additional sampling effort. Non-sterile tools and receptacles are acceptable for DNA analysis of the coral host and Symbiodiniaceae but are not desirable for certain DNA analyses. However, if total Symbiodiniaceae community diversity is being assessed, care should be taken to avoid cross-contaminating specimens by wiping and/or rinsing tools and receptacles between samples. Aseptic sampling techniques and aseptic equipment (e.g., wearing nitrile gloves and storing each sample in separate sterile transport receptacles) are optimal when characterizing microbial communities, and choosing to proceed without using those techniques significantly limits the reliability of DNA-based microbial community analysis. DNA analyses do not require large or high biomass samples, as the optimal sample should have a surface area of 1–2 cm^2^ for most DNA analyses, or 1–3 polyps if sampling via syringe extraction (e.g., (Kemp, Fitt & Schmidt, 2008; Correa et al., 2009)). Although DNA is stable for extended periods of time, such samples should be fixed in preservative and/or frozen at −80℃ as quickly as possible. The amount of time between sampling and preservation of DNA can be upwards of one hour if stored in seawater or on ice but minimizing the length of this interim period will benefit most analyses, particularly those focused on microbial communities. Following the initial preservation of the sample, most storage options below −20℃ are acceptable (e.g., −40℃) for maintaining the quality of DNA in samples. When freezing, samples should be rapidly frozen in liquid nitrogen as soon as possible following transport on ice. However, freezing at −20℃ can be acceptable if other options are unavailable. In most instances, storage of the samples at 4℃ or room temperature for any extended period is not desirable (Rubin et al., 2013). The addition of liquid preservatives or salt buffers may help maintain the quality of the DNA in the sample if it is being preserved for months to years prior to analysis. Several preservatives (e.g., formaldehyde) have yet to be thoroughly tested in terms of their ability to maintain quality of coral, Symbiodiniaceae, and coral-associated bacteria or virus DNA. The utility of these solutions for short- and long-term storage are therefore considered unknown. If a preservative is not immediately used following sample collection, the samples can be transported in a cooler with ice for up to an hour but longer amounts of time may lead to some changes in the microbial community composition (Rubin et al. 2013).

*Short-Term Preservation of Samples*: There are several preservatives and storage methods that are optimal for DNA analyses, including concentrated ethanol (as high as possible, up to 95%), salt buffers including, dimethyl sulfoxide (DMSO) (Gaither et al., 2011; Gray, Pratte & Kellogg, 2013; Hernandez-Agreda, Leggat & Ainsworth, 2018), RNA/DNA Shield, paraformaldehyde (PFA) (Ainsworth et al., 2015) to phosphate buffered saline (PBS) (Hernandez-Agreda, Leggat & Ainsworth, 2018; Greene et al., 2020), and freezing at −80℃. Storage at −20℃, 4℃, or room temperature tends to be acceptable if combined with one of the previously listed preservatives (Dawson, Raskoff & Jacobs, 1998).

*Long-Term Preservation of Processed Samples*: Following DNA isolation, any remaining product not used for the present analyses should optimally be archived at −80℃ with few to no freeze-thaw cycles to maintain the quality of the DNA. Samples most likely could be stored long-term at −40℃, although that has not been explicitly tested on coral holobiont DNA. Variability in DNA extraction and amplification success is common among different species of cnidarians, so the testing of preservation methods for the specific species being studied is advised (Dawson, Raskoff & Jacobs, 1998; Gaither et al., 2011). While stored DNA should remain usable for several years, confirming the quality of the DNA is important regardless of storage duration because significant DNA degradation can occur within the first 24hrs following preservation in a suboptimal preservative for some corals (Gaither et al., 2011). Several freeze-thaw cycles over one year may degrade the quality of a DNA sample, while a sample stored for 5–10 years with minimal disruption could be in good condition (Dawson, Raskoff & Jacobs, 1998; Shao, Khin & Kopp, 2012).

*Archiving Extra Coral Fragments*: Additional material from the original sample to be archived for future work can be stored as an unprocessed fragment or in the form of ground coral or airbrushed blastate. Such samples may be stored for more than 10 years if maintained at −80℃ with minimal freeze thaw cycles; sterile glass or plastic receptacles are acceptable for all downstream DNA-based analyses. Preservatives like RNA/DNA Shield or other salt/salt-saturated buffers are also acceptable for long-term storage if paired with −80℃ to −40℃ temperatures.

RNA-Based Work

*Sample Collection*: Coral specimens for RNA-based analyses should be collected using aseptic techniques to maximize the number of analyses that can be completed without additional sampling effort, and to minimize sample contamination and degradation by foreign RNA and RNAses, etc. Aseptic sampling techniques and sterile equipment are optimal when characterizing RNA viruses from corals or their symbionts. Small amounts of tissue are potentially sufficient for RNA-based analyses, particularly for phylogenomics. However, if sample degradation or changes of gene expression (due to tissue disruption) are a concern, it may be advantageous to collect a larger initial fragment in the field, then sub-sample and preserve a small undisturbed portion. For gene expression work, sample collection should be standardized to specific hours of the day as diel changes in expression patterns are well documented (e.g., (Levy, Dubinsky & Achituv, 2003; Levy et al., 2007; Hemond & Vollmer, 2015; Wright et al., 2019)). Care should be taken to preserve samples as quickly as possible to avoid alteration of gene expression patterns and/or degradation (note that temporary storage temperatures and times are significantly more restrictive for RNA than DNA in Table 1).

*Short-Term Preservation of Samples*: Samples for RNA-based analyses should be stored using sterile tools and receptacles, when possible, as non-sterile equipment will lead to contamination and/or RNA degradation. RNA-based short-term preservation closely mirrors that for DNA-based analyses. For many preservatives in Table 1, there are data available for other model systems, but no literature available for coral holobionts on the efficacy or caveats of a given preservative (e.g., formalin, glutaraldehyde, methanol).

*Long-Term Preservation of Processed Samples*: The main difference in long-term preservation approaches for RNA- versus DNA-based downstream analyses is that for RNA, it is preferable to rapid-freeze whole tissue samples (i.e., intact coral fragments) to −80℃ without freeze-thaw cycles, rather than to preserve partially processed tissues (e.g., blastate) long-term.

*Archiving Extra Coral Fragments*: Considerations and caveats for archiving extra coral fragments for RNA-based analyses closely follow the information above (for RNA-based short-term and long-term processed sample preservation and storage). Since RNA is more sensitive to degradation than DNA (Ji et al., 2017), it may be difficult to achieve sufficient RNA yields from samples that have been stored for years or decades, or in suboptimal preservatives.

Protein-Based Work

*Sample Collection:* The optimal method for collection of specimens for protein analyses is to use aseptic techniques (clean collection tools and storage containers) and to get the samples frozen as quickly as possible. Non-sterile tools and containers are acceptable, though sterile practices are optimal if samples are to be analyzed for bacteria, viruses or other microbes; though we are not aware of any studies to date explicitly targeting non-Symbiodiniaceae microbes of corals for protein analysis. Small amounts of tissue (~1cm^3^) are potentially sufficient for protein analysis, though it is common practice to collect a larger fragment (2-3 cm^3^) and sub-sample during the protein extraction phase.

*Short-term Preservation of Samples:* The optimal approach would be to flash freeze the samples as soon as possible after collection (e.g., on the boat or once returned to shore), though keeping samples on ice packs that have been incubated in an ultra-cold freezer (−40°C or below) is a potential alternative if liquid nitrogen is not available. Short-term storage in a cooler on regular ice is acceptable up to 1 hour, as is short-term freezing at −20°C. It is unknown how much protein degradation occurs on ice or at −20°C for whole coral fragments, so longer times may be acceptable if high quality protein is able to be extracted from samples that were not frozen in < 1hr. The biggest difference with protein preservation compared to nucleic acid analyses is that we are not aware of any field preservation buffers that are reliable for stabilizing proteins and inactivating proteases in an intact coral sample, thus freezing is the optimal approach.

*Long-term Preservation of Processed Samples:* Rapid-freezing of remaining fragments of whole tissue (i.e., intact coral fragments) to −40 to −80°C without freeze-thaw cycles is optimal, though rapidly frozen tissue blastate is also acceptable and may be optimal if subsequent protein activity assays are the desired end point analysis. Alternatively, extracted protein may be preserved frozen (at −20°C or below) in a reliable protein extraction buffer or lyophilized and stored frozen as a solid. Slow degradation of samples is possible, though the authors have experience using frozen protein extracts up to 2 years post-extraction with high quality yields. A total protein gel or other suitable quality assessment and control (QA/QC) is recommended in general, but particularly for samples that have been stored under less-than-optimal conditions or for extended (>2 years) periods of time.

*Archiving Extra Coral Fragments:* Considerations and caveats for archiving extra coral fragments for protein-based analyses closely follow the information above (for protein-based short-term and long-term processed sample preservation and storage), including the recommendations for QA/QC to verify protein integrity.

Epigenetics

Epigenetic research is relatively new, and thus does not have the same wealth of previous studies from which to draw general recommendations as exists for DNA, RNA, and protein work (Allis et al., 2015). Depending on the type of epigenetic research being pursued, current protocols fall largely in line with the DNA, RNA, or protein analyses sections. In general, methylation approaches (e.g., DNA immunoprecipitation sequencing (MeDIP-seq), EpiRAD, RRBS, etc.) tend to align with recommendations typical of the DNA-based analyses above, whereas RNA, nucleosome or genomic architecture (e.g., histone or chromatin configuration) approaches (e.g., miRNA, ATAC-seq, ChIP-seq, 3C, etc.) tend to fall closer to those for RNA or proteomics outlined below.

*Sample Collection*: As with the nucleic acid sections, the use of aseptic technique is not absolutely critical for these methods but if used will allow for expanded downstream applications. Small amounts of tissue are generally sufficient, again in keeping with the DNA and RNA recommendations. While it is possible to perform some methods using DNA preserved in the least stringent methods outlined above, epigenetic studies tend to be most informative when multiple classes of molecules (DNA, RNA, and protein) are collected and processed from the same sample. Although it may not be desirable or possible to process all these classes simultaneously, the ability to return to the same specimens and expand on previous studies could provide invaluable insights into epigenetic mechanisms. Samples therefore can ideally be collected in a manner consistent with DNA, RNA, and protein recommendations to allow for downstream epigenetics work.

*Short-Term Preservation of Samples*: Specific applications will have differing requirements for sample preservation, but the number of approaches available to explore for epigenetic studies are limited by the requirements of the most stringent aspect of the molecules under study. For DNA methylation, short-term storage at room temperature in a suitable preservative is possible, but for miRNA or ATAC-seq, such treatment would destroy the sample. Frequently, multi-molecule studies will require rapid-freezing coral fragments of moderate size (1–2 cm^2^) to allow for multiple analyses, and storage at −80℃ or below with subsampling of the frozen fragment for each of the analyses performed.

*Long-Term Preservation of Processed Samples*: As with the short-term preservation above, the storage method depends on the approach. If the sample is a DNA extract for methylation studies only, the sample could be stored as outlined in that section above. For multi-molecule studies, samples should ideally be rapid-frozen and continue to be stored at −80℃ without freeze-thaw cycles.

*Archiving Extra Coral Fragments:*  To maintain the quality and integrity of the range of molecules containing epigenetic information for coral samples, ideally the sample would be rapid-frozen and stored at −80℃ without thawing. The maximum time for which cryopreserved samples remain useful is unknown, particularly for corals, in which only a handful of epigenetic studies have been performed to date (e.g., (Putnam, Davidson & Gates, 2016; Torda et al., 2017; Dimond & Roberts, 2020; Rodríguez-Casariego et al., 2020)). It is expected that samples are likely to be useful for a couple of years, limited to the most sensitive of the molecule classes. 

Metabolomics

The application of metabolomics has proved especially powerful for elucidating the metabolic basis of the cnidarian-dinoflagellate symbiosis and how it responds to thermal stress (Hillyer et al., 2017, 2018; Williams et al.). Moreover, given the especially close link between the metabolite composition and an organism’s physiology and health, it is thought that metabolomics has greater potential for environmental monitoring and provides a more direct measure of organism functioning than other Omics platforms such as transcriptomics and proteomics (Bahamonde et al., 2016). However, as with epigenetics, its application to study corals is still relatively new, and sample collection and preservation techniques are still being optimized (Matthews et al., 2022). Optimal sample preservation can depend on whether the profiling is untargeted or targeted, as well as the analytical platform destination, including proton-nuclear magnetic resonance spectroscopy (^1^H-NMR), liquid chromatography-mass spectrometry (LC-MS), and/or gas chromatography-mass spectrometry (GC-MS). While discussion of specific approaches is beyond the scope of this manuscript, basic in-field and in-lab techniques for sample collection, preservation and storage are more generalizable and a conservative (platform- and metabolite-wide) approach can be described.

*Sample Collection*: When examining variable responses, like in the case of coral bleaching, the time between sample collection and preservation (i.e., time taken to quench metabolism) is critical to capture the original response. The goal should be to preserve a sample as quickly as possible after collection, preferably via rapid freezing in liquid nitrogen, to prevent changes in metabolite composition due to sample degradation (Mushtaq et al., 2014). The time of collection period should also be taken into consideration as diurnal processes in both the host and algal members will lead to radically different metabolic profiles depending on the time of day the sample is taken. For example, circadian rhythms for both members and daylight vs. nighttime involve different metabolic processes affecting the analytical outcome. The target metabolites should also be considered prior to sampling as the downstream preservation and analysis might differ depending on the metabolite class of interest (e.g., carbohydrates or lipids). Final biomass is also a critical consideration, particularly for coral bleaching experiments, as algal symbiont density is typically reduced, and untargeted metabolomic analyses of Symbiodiniaceae fractions will require sufficient biomass for metabolomics platforms (~15mg dry weight).

If rapid preservation is not possible, all samples should be treated equally throughout the whole sampling protocol and have a similar sampling to freezing time window. This will enable sample preservation homogeneity to correct for any possible degradation artifacts. The Metabolomics Standards Initiative (Fiehn et al., 2007; Sumner et al., 2007) states that the preferred minimum sample size is five replicates (n=5).

*Short-Term Preservation of Samples*: Upon collection, the sample should be immediately rapid frozen in liquid nitrogen for optimal results although some other preservation methods may work with caveats (see Table 1). Ideally, samples will be frozen at −80℃, and freeze-thaw cycles avoided, in light-blocked containers until processing.

*Long-Term Preservation of Processed Samples*: Samples should always be maintained at −80℃ temperatures, preferably in light-blocked containers.

**Physiology Methods:**

Chlorophyll and other pigments

*Collection, Sacrifice and Preservation:* Samples for chlorophyll quantification can be collected from field or experimental samples using sterile or non-sterile equipment. Whole coral fragments, tissue samples or planulae are acceptable and generally require 1 cm^2^ or more of coral tissue (however, more may be required if samples are significantly bleached). Rapid freezing and short-term storage with dry ice is acceptable, but CO_2_ gas can create acidic conditions that may lead to some degradation (Roy et al., 2011). Both liquid nitrogen and ultra-cold freezing are suitable for rapid-freezing samples intended for pigment analysis, including accessory pigments that may rapidly convert under certain light levels (Southerland & Lewitus, 2004; Warner & Berry-Lowe, 2006). Sample storage in liquid nitrogen (−196℃) or ultra-cold freezing (−80℃) are commonly employed for long-term storage prior to extraction (Roy et al., 2011), and algal samples stored on filters at −80℃ have remained stable for up to one year. It is also recommended to store samples in air-tight packaging with as much air evacuated as possible to prevent oxidation (Roy et al., 2011).

*Processing*: Extraction in 100% acetone or acetone:water (90:10) are historically the most common solvent mixtures for Symbiodiniaceae, followed by spectrophotometry with the calculations of Jeffrey and Humphrey (Jeffrey & Humphrey, 1975). Fluorescence-based measurements are also possible and typically more sensitive to low concentrations (Holm-Hansen & Riemann, 1978). Acetone extracts from other microalgae tend to show minimal degradation when stored for <20 days at −15℃, and total chlorophyll and carotenoid degradation rates are ~ –0.2% d^-1^(Hooker 2005). In addition to acetone, alternative methods, such as extraction in methanol and spectrophotometry are also available (Porra, Thompson & Kriedemann, 1989; Hoadley et al., 2019). For Symbiodiniaceae extracted at room temperature from the scleractinian coral *Pocillopora capitata*, both acetone and methanol-based extraction methods achieved peak fluorescence within 1 hour, indicating complete extraction (Holm-Hansen & Riemann, 1978). Longer extraction times of up to 12 hours are also acceptable but may be best at lower temperatures (−20℃). While not as common, lyophilization (freeze-drying), immediately followed by extraction, is also an effective method for pigment extraction and in some cases improves the chlorophyll extraction efficiency (van Leeuwe et al., 2006), including in Symbiodiniaceae from soft corals (Pupier, Bednarz & Ferrier-Pagès, 2018).

Mycosporin-Like Amino Acids (MAAs)

*Collection, Sacrifice and Preservation:* Samples for mycosporin-like amino acid (MAA) determination can be collected from field or tank-based experiments using sterile or non-sterile tools. Whole coral fragments (skeleton + tissue), tissue, or Symbiodiniaceae samples are acceptable. MAAs can also be determined from mucus, as it has already been done for fish (Reverter et al., 2018), or for corals (Teai et al., 1998). Planulae can also be analyzed for MAAs (Zhou et al., 2016). Coral surface area of ~2cm^2^ or greater is needed for the quantification and identification of MAAs. The best practice for sample storage is to rapid freeze in liquid nitrogen and transfer to a −80℃ freezer. Short-term storage (immediately after collection) in cooler ice or in bags or tubes filled with seawater and kept at 4℃ on the boat are also acceptable for up to 4–6 hours as long as temperature is monitored and samples are preserved from light (Corredor et al., 2000). Additionally, coral tissue, Symbiodiniaceae, or larvae can be kept at −20℃ for a week or freeze-dried and kept at −80℃ for long-term storage. Preservatives such as formalin, ethanol, peroxide, and bleach should not be used for these samples, as such chemicals can interact with those used for the extraction of MAAs.

*Storage of Processed Samples:* Typically, there is little archiving of processed material because MAA assays generally consume the specimen.

*Long-Term Storage:* For long-term storage of samples before analysis it is optimal to preserve samples at −80℃.

Lipid, Protein, Carbohydrates, and Biomass Assessments

*Collection, Sacrifice and Preservation:* Samples collected for energy reserve quantification can be collected from field or tank-based experiments using sterile or non-sterile tools. Whole coral fragments (skeleton + tissue), tissue samples (airbrushed or water-pik), mucus, or larvae (planula) are acceptable. Coral surface area of ~1cm^2^ or greater is needed for quantification of each energy reserve though new microplate methods are in development that may allow for smaller fragment sizes in future. The best practice for sample storage is to rapid freeze in liquid nitrogen or dry ice or at −80℃ and store in a −80℃ freezer. While storage at −20℃ is also acceptable, storage at refrigerator or room temperature is not advised. In the absence of immediate freezing capacity, short-term storage (immediately after collection) on blue ice (cooler + ice) for up to 1 hour or storage in live-well buckets or bags on the boat or during transportation is also acceptable for up to 4-6 hours as long as water is changed often and temperature is monitored. Additionally, corals can be shipped to the laboratory live using wet paper towels and sealed plastic receptacles or wet bubble wrap. This live transport method affords roughly 48 hours of viability but can have significant impacts on energy reserves. Preservatives such as formalin, methanol, ethanol, peroxide, and bleach should not be used for these samples, as such chemicals can damage cells, leach energy reserves, and alter energy quantification. It is important to acknowledge that lipid, protein, carbohydrate, and biomass values may be underestimated when using airbrushed tissue, as a significant proportion of organic tissue resides within the skeletal organic matrix (Conlan, Rocker & Francis, 2017). Though lipid, protein, and carbohydrates can be standardized to surface area, standardization to ash free dry weight is more robust for comparisons among coral species and specimens with different tissue thicknesses (Edmunds & Gates, 2002). Biomass is standardized to surface area.

Total Soluble Lipids

One-gram wet weight of ground coral (whole tissue plus skeleton) is suitable for lipid extraction. Ideally samples are freeze-dried prior to analysis. A similar wet weight of air-brushed tissue blastate or collected mucus can be used as well, though the use of these methods may result in lipid concentrations (and classes) that differ from methods that utilize whole, ground coral fragments. Lipid extractions should be extracted using 2:1 Chloroform: Methanol (Hara & Radin, 1978; Harland et al., 1991; McLachlan, Munoz-Garcia & Grottoli, 2020). Notably, a lipid extraction method that utilizes 2:1 Dichloromethane (DCM): Methanol (MeOH), which has been successful in extracting lipids from other organisms (Christie & Han, 2010) has proven unsuccessful and unreliable for at least some species of corals (Baumann et al., 2014). Lipid concentrations can be quantified via weight (McLachlan, Munoz-Garcia & Grottoli, 2020) or through colorimetric assays in microplates (Cheng, Zheng & VanderGheynst, 2011). It should be noted that ideally extracted lipids should be stored in air-tight receptacles. Amber glass receptacles are useful to prevent oxidation and photodegradation, but frozen lipid samples can also be stored in plastic receptacles. Following lipid extraction into solvents, glass storage receptacles are required to avoid reactions of solvents with plastics. It is recommended that all glassware be washed with non-phosphate soap and pre-baked (suggested method: (McLachlan, Munoz-Garcia & Grottoli, 2020).

*Storage of Processed Samples:* If lipids are extracted via chloroform:methanol and dried (to determine lipid weight) they can be stored for additional analyses (e.g., lipid class determination via high performance liquid chromatography (HPLC) or Iatroscan) by resuspending the dried lipids in chloroform (Christie, 2003). Lipids should be stored in sealed amber glass vials in the dark and are viable for additional analyses for multiple years (<10). Acceptable storage methods include −80℃ freezer and liquid nitrogen / cryopreserved are likely acceptable though not tested.

Proteins

Half a gram of ground whole coral (wet weight) or 0.5–1.0ml of tissue blastate can be used for most protein extractions. Similar wet weights or volumes are likely suitable for collected mucus. In all cases slightly higher amounts of weight wet or volume are recommended to allow for replicate sampling, method testing, and as a buffer should the protein content of your preferred sample type be low. Soluble proteins (host, *Symbiodiniaceae*, or holobiont) can be quantified using a colorimetric method (McLachlan et al., 2020).

*Storage of Processed Samples:* There is no archiving of processed materials because protein assays using the colorimetric BSA protein method render sample unusable for additional downstream analyses

Carbohydrates

Whole coral samples of ~1cm^3^ or 1g are recommended for carbohydrate quantification. More sample may be required for bleached corals. Carbohydrate concentrations can be measured following phenol and sulfuric acid extraction using a colorimetric procedure (DuBois et al., 1956). Smaller coral sample amounts may be utilized for more modern, microplate-based colorimetric carbohydrate protocols (*sensu* (Masuko et al., 2005)). However, 1cm^3^ is still recommended to allow for triplicate analysis of each sample.

*Storage of Processed Samples:* There is no archiving of processed materials because carbohydrates assays using the colorimetric method render sample unusable for additional downstream analyses.

Biomass

One gram of ground whole coral (wet weight) can be used for most ash-free dry weight biomass determinations. Similar wet weights are likely suitable for airbrushed tissue blastate or collected mucus, but slightly higher amounts of wet weight are recommended to allow for replicate sampling, method testing, and as a buffer should the biomass content of the preferred sample type be low. It is important to acknowledge that biomass values may be underestimated when using airbrushed tissue, as a significant proportion of organic tissue resides within the skeletal organic matrix (Conlan, Rocker & Francis, 2017), and this is also true for protein, lipid, and carbohydrate analyses. Tissue biomass (host, *Symbiodiniaceae*, or holobiont) can be quantified by drying coral material to a constant weight (60℃ for 24 hrs) and burning it (450℃ for 6 hrs) to yield the ash-free dry weight (McLachlan, Dobson & Grottoli, 2020).

*Storage of Processed Samples:* There is no archiving of processed materials because biomass analyses completely consumes the sample leaving no materials for additional downstream analyses (McLachlan, Dobson & Grottoli, 2020).

Stable Isotopes in Tissues (Whole, Host, and Symbiodiniaceae δ^13^C and δ^15^N)

Natural abundance stable isotopes in coral tissue samples have been used to determine the proportion of heterotrophy vs photoautotrophy in corals (Muscatine, Porter & Kaplan, 1989; Rodrigues & Grottoli, 2006; Grottoli, Tchernov & Winters, 2017; Price et al., 2021; Ferrier-Pagès et al., 2021), the trophic status of corals (Conti-Jerpe et al., 2020; Price et al., 2021) and to determine the proportionate contribution of various food sources to coral tissues (Price et al., 2021). Isotopic enrichment experiments and pulse-chase isotope labeling using both δ^13^C and δ^15^N have successfully identified pathways of C and N acquisition and allocation within corals (Piniak, Lipschultz & McClelland, 2003; Hughes et al., 2010; Hughes & Grottoli, 2013; Baumann et al., 2014; Tanaka et al., 2015; Ezzat et al., 2017; Pupier et al., 2021).

Coral tissue is removed from the skeleton by air-brushing or water-piking. The resulting blastate is then processed for isotopic analysis of the whole coral, or the blastate is further processed to separate the coral host from the endosymbiont via a series of sonication and centrifugation steps. Preparation for stable isotopic analysis can be completed for dried down whole tissue, host tissue, and Symbiodiniaceae tissue (Price et al., 2020). Other methods are similar, but tissue material may be loaded onto a pre-burned GFF filter (Rodrigues & Grottoli, 2006). However, GFF filters necessitate a larger tin for packing the samples, reducing the number of samples that can be analyzed in a single run, and clog the combustion column resulting in more frequent column cleaning and higher analytical costs.

Isotopes and Element/Calcium in Coral Skeletal Material

Stable isotopes and element/calcium ratios in coral skeletal samples are widely used to reconstruct past climate and environmental changes at annual to monthly resolutions (Grottoli & Eakin, 2007; Felis, 2020), although a reliable geochemical identification of bleaching events in skeletal records is still in its infancy (D’Olivo & McCulloch, 2017). Skeletal δ^13^C has been used to reconstruct the autotrophy-heterotrophy contribution of carbon in the coral skeleton (Felis et al., 1998; Grottoli & Wellington, 1999), seasonal changes in light levels (Grottoli, 2002), and the ^13^C Suess effect as a function of anthropogenic CO_2_ emissions (Swart et al., 2010). Skeletal δ^18^O (Wellington, Dunbar & Merlen, 1996; Boiseau et al., 1998; Quinn et al., 1998), Sr/Ca and U/Ca (Felis et al., 2009), Li/Ca and Li/Mg (Hathorne et al., 2013), Sr-U (Alpert et al., 2017) and clumped isotopes (Saenger et al., 2012) have been used to reconstruct the temperature and hydrology of the surface ocean (Felis, 2020).

Skeletal boron isotopes (δ^11^B) and boron/calcium (B/Ca) are used to determine the effects of ocean acidification on coral calcification (McCulloch et al., 2017) and to reconstruct the history of ocean pH (Hemming & Hanson, 1992; Hönisch et al., 2004). Nitrogen isotopes (δ^15^N) in skeleton-bound organic matter have been used to provide information about the oceanic nitrogen cycle and the influence of anthropogenic nitrogen on the open ocean (Wang et al., 2018). Skeletal barium isotopes (^138/134^Ba) and cadmium (Cd/Ca) have been suggested as a proxy for oceanic barium cycling (LaVigne et al., 2016; Liu et al., 2019) and upwelling (Shen, Boyle & Lea, 1987), respectively. The combination of element/Ca and skeletal boron (δ^11^B) isotope records have been successful at detecting the response of coral calcification and calcifying fluid to thermally induced bleaching stress (D’Olivo & McCulloch, 2017).

*Sample Collection:* Coral skeletal cores are collected using underwater pneumatic or hydraulic coring devices. Coral skeletons of ramets and whole colonies are also collected, depending on the study. Aseptic techniques are not necessary. Coral tissue is removed with an airbrush or water-pik. Coral cores, colonies, and fragments are cut into longitudinal 1cm thick slabs along the major axis of growth and dried thoroughly, preferably at 60℃ for several days if possible. Coral skeleton sub-samples are collected by hand using a micro-milling or rotary tool under a dissecting microscope for monthly to annually resolved analyses, and with high-precision micromilling/microdrilling for annually to monthly resolved analyses. All samples are collected along the major axis of growth (Giry et al., 2010). The resulting skeletal powder is then processed for isotopic and elemental analysis using specific preparation steps depending on the various analytical methods. For δ^13^C and δ^18^O analyses, care must be taken not to chemically clean samples prior to stable isotopic analyses as this can cause uncorrectable isotopic fractionation (Grottoli et al., 2005). High-pressure water is sufficient to remove debris from the surface prior to drying and drilling a skeletal core. However, element/Ca analyses typically involve significant chemical cleaning steps (Matthews, McDonough & Grottoli, 2006). An alternative to drilling is direct “non-destructive” analysis of coral skeletal slabs for element/Ca ratios by Laser Ablation Inductively Coupled Plasma Mass-Spectrometry (LA-ICP-MS) (Matthews, McDonough & Grottoli, 2006; Hathorne et al., 2011) and X-Ray Fluorescence (XRF) scanning (Ellis et al., 2019).

*Short-Term, Long-term, and Archiving of Skeletal Samples:* Coral cores, whole fragment or colony skeleton, and ground coral skeletal powder can all be stored dry at room temperature or at 4℃ indefinitely. The most important is that the skeletal material be initially dried following collection in a drying oven at 60℃ until completely dry, then stored dry so that mold does not grow on the skeleton.

**Microscopy & Imaging** **Methods**:

Tissue and Skeleton Ultrastructure

While many features of corals undergoing bleaching can be viewed using visible light, others require methods that can resolve smaller external and internal features such as the structure and dynamics of cell membranes and organelles as well the intracellular and extracellular placement and morphology of microbial symbionts, viral particles, or proteins. In these cases, techniques such as scanning and transmission electron microscopy (EM) can be used. Although the varieties of staining procedures for EM are beyond the scope of this manuscript, basic in-field and in-lab techniques for preservation and storage methods of coral samples are more generalizable and have been published on extensively in the past.

*Sample Collection:* Coral tissues collected for scanning and transmission electron microscopy (SEM and TEM, respectively) should be fixed and never frozen (with the exception of cryo-EM, which requires special preparation prior to freezing, see Box 1). Samples collected for skeletal analyses do not require fixation and can be stored at room temperature. Depending on the application, tissues should be fixed immediately upon collection (e.g., Nano-

Secondary Ion Mass Spectrometry (Nano-SIMS), immunolocalization, microbiome work). Fixing tissues with EM-grade reagents is optimal to avoid background fluorescence. Sterile tools are only required for microbiome applications but samples exploring other aspects of the holobiont need not use aseptic techniques.

*Short-Term Preservation of Samples:* No preservation is required for skeletal samples. However, preservation methods for tissues vary, but typically consist of a combination of paraformaldehyde and glutaraldehyde and some salt buffer such as phosphate buffered saline or sodium cacodylate (Price & Peters, 2018; Greene et al., 2020). Fixed tissues are best stored in glass receptacles at 4ºC.

*Sample Processing:* Tissue removal from skeletal samples is typically done by immersion in dilute bleach solution to remove all organic material. Skeletons are coated with gold-palladium prior to imaging (Tambutté et al., 2007). Fixed tissues can be fractured or mounted directly onto EM grids for SEM. For TEM of tissues, samples must first be decalcified typically in high molar EDTA and must then be sectioned using a microtome, mounted, dehydrated and stained (Correa et al., 2016). No further processing is required unless immunolocalization is desired.

*Long-Term Preservation of Processed Samples:* Once tissues have been mounted on EM grids either whole (SEM) or sectioned (TEM) and imaged, they can be stored at room temperature and reimaged at a later date. Some damage to tissue sections can occur during TEM imaging. Skeletal samples can be stored indefinitely at room temperature for future use.

*Archiving of Unprocessed Samples:* Tissues embedded in blocks or cryopreserved can be stored indefinitely; skeletons can be stored indefinitely at room temperature ideally with low humidity.

Histology: Gross Morphology

Histological techniques can provide a wealth of information about coral bleaching at the cell and tissue levels. Histology provides evidence on several coral aspects such as cell structure and integrity, presence of endosymbionts (Symbiodiniaceae and other microorganisms) and presence of diseases and pathogens (Bythell et al., 2002; McClanahan et al., 2004; Work & Meteyer, 2014; Gierz et al., 2020). Important efforts have been made for the last four decades to optimize coral sampling, preservation, and observation for histological investigations, leading to numerous valuable protocols (Hayes & Bush, 1990; Brown, Le Tissier & Bythell, 1995; Greene et al., 2020). Also, the difference in terms of morphology and proteins and lipids compositions induce the use of different protocols among coral species, life stage, endosymbiont type (Rinkevich & Loya, 1979; Harii et al., 2009). While the diversity in laboratory techniques is beyond the scope of this manuscript, universal basic procedures are highlighted here.

*Sample Collection:* Coral tissues collected for histological investigations should be fixed immediately (or transported briefly on ice) upon collection and never frozen in order to preserve cell integrity for future observations. Samples can be collected without concern for sterility and require at least 1cm^2^ of adult coral tissue for histological analysis or entire larvae.

*Short-Term Preservation of Samples:* Short-term preservation type and time vary among studies, but mostly consist of a combination of formalin/formaldehyde with seawater, or paraformaldehyde/glutaraldehyde with phosphate-buffered saline solution (PBS) (Hayes & Bush, 1990; Brown, Le Tissier & Bythell, 1995; Bythell et al., 2002; McClanahan et al., 2004; Harii et al., 2009). The relative proportions of each preservative and the time of preservation differs slightly among studies but preserving in 10% formalin in seawater is common practice. EM-grade fixatives are preferred for applications requiring fluorescence imaging (see next section); however, non-EM grade reagents are acceptable for downstream applications that do not require fluorescence (e.g., hematoxylin and eosin staining).

*Sample Processing:* For tissue observations, coral samples are decalcified in a short-term preservative, but sometimes the use of formic acid, ascorbic acid, or ethylenediaminetetraacetic acid (EDTA) can help the decalcification process (Hayes & Bush, 1990). The samples are then dehydrated using a graded series of ethanol (EtOH) and then embedded in commercial resin. Sections are made using a microtome and slices mounted onto a glass slide (dye can be added) for observation of cells and/or tissues under photomicroscope (Hayes and Bush 1990; Brown et al. 1995; Bythell et al. 2002).

*Long-Term Preservation of Processed Samples:* Histology processing is not destructive, allowing the slices to be stored for years and be re-used for further observations. The slices need to be stored in a dark, cool, and dry location. Ideally, samples need to be stored in specialized boxes that protect slides from dust deposition.

*Archiving of Unprocessed Samples:* The unprocessed samples in the preservative solutions can be stored for a short (days to weeks) time. Once transferred to ethanol, fixed tissues can be stored at 4℃ for months to years. Alternatively, samples embedded in resin can be archived for years for future sections if stored in a dark, cool, and dry location.

Histology: Localization of Nucleic Acids and Proteins

*Sample Collection:* Coral tissues collected for localization of nucleic acids or proteins should be fixed immediately upon collection (or transported briefly on ice) using EM-grade reagents and kept at 4℃ but never frozen. Samples collected for florescence in situ nucleic acid hybridization (FISH) require molecular grade reagents and equipment. Sterile tools are only required for microbiome applications. At least 1cm^2^ of adult coral tissue for histological analysis or entire larvae is sufficient.

*Short-Term Preservation of Samples:* Tissue fixation protocols vary, but mostly consist of a combination of paraformaldehyde with or without glutaraldehyde in a phosphate-buffered saline solution. The relative proportions of each preservative and the time of preservation differs slightly among studies, but fixation is generally conducted in less than 24 hours from collection at 4ºC. EM-grade fixatives are preferred for applications requiring fluorescence imaging; non-EM grade reagents are acceptable for downstream applications that do not require fluorescence (e.g., colorimetric immunoperoxidase development).

*Sample Processing:* Coral samples are decalcified in a short-term preservative, but sometimes the use of formic acid, ascorbic acid, or EDTA can help the decalcification process (Hayes & Bush, 1990). The samples are then dehydrated using a graded series of ethanol, xylene (or xylene substitutes), and then embedded in commercial resin (e.g., paraffin wax). Sections are made using a microtome and slices mounted onto a glass slide, wax removed, and tissues rehydrated. It is recommended to process tissue sections the same day, either with nucleic acid probes, antibodies, and/or nucleic acid stains (e.g., 4′,6-diamidino-2-phenylindole (DAPI); Hoescht). If slide will not be hybridized (antibodies or FISH probes) within 24–48 hours of sectioning, the slide may be stored in a sealed receptacle in the freezer at −20℃ for up to 2 weeks (Wakai et al., 2014). It is recommended to view and image slides within a few days of hybridization.

*Long-Term Preservation of Processed Samples:* Formalin-fixed samples probed with nucleic acids (e.g., FISH) can be re-used once they are observed, in which case the probe is removed with formamide and the slide can then be stored for future re-probing at −20℃ or −80℃ for at least 100 days. Fixed cell suspensions should be stored in cryovials in the freezer (−20℃) (Wakai et al., 2014). Labeled tissue sections can be stored for further observations and imaging. FISH slides can be stored for up to 1 year at −20℃ in the dark (Alamri, Nam & Blancato, 2017). Immunochemistry slides can be stored at −20℃ to 4℃ in the dark for years. Ideally, slides need to be stored in specialized boxes that protect them from dust deposition and light.

*Archiving of Unprocessed Samples:* Fixed tissue samples can be stored in 100% ethanol at 4℃ for years (Schimak, Toenshoff & Bright, 2012). Tissues embedded in resin can be archived for years for future sectioning if stored in a dark, cool, and dry location.

Skeletal Imaging (CT Scanning, X-RAY, Dyes & XRF Scanning)

*Sample Collection:* Skeletal imaging techniques are commonly applied to skeletal cores but are also applicable to coral fragments. Skeleton samples must be rinsed thoroughly immediately upon collection to remove seawater and avoid salt contamination.

*Short-Term Preservation:* Prior to analysis, all skeletal imaging techniques require that samples are cleaned of seawater and dried. Ideally, samples should not be stored in chemical preservatives to avoid dissolution of the skeleton, precipitation of new minerals on the sample, or any other alteration to the skeletal integrity. However, Computed Tomography (CT) scanning and X-Ray analysis have been applied to skeleton samples previously frozen or stored in ethanol, and these techniques (at least when investigating features on the mm or cm scale) are not highly sensitive to minor dissolution/precipitation on the micron scale.

*Sample Processing:* The processing procedures for skeletal imaging vary among techniques. The benefit of CT scanning is that entire samples (e.g., cores) can be scanned intact, without any alterations. Conversely, X-ray and x-ray fluorescence (XRF) require cores to be sliced, while analysis of dyes typically requires the preparation of polished sections embedded in epoxy.

*Long-Term Preservation of Processed Samples:* Processed skeletal samples should be stored in a cool, dry location. The samples must be entirely dry, and ideally should be wrapped in plastic to prevent mold growth or dust deposition. Fluorescent dyes should be stored in the dark to avoid slow photo-bleaching of the dye.

*Archiving of Unprocessed Samples:* Skeletal cores and coral fragments can all be stored dry at room temperature or at 4℃ indefinitely. The most important is that the skeletal material be initially dried following collection in a drying oven at 60℃ until completely dry, then stored dry so that mold does not grow on the skeleton. For sliced cores, the same long-term preservation noted above applies to the unprocessed halves. It may be beneficial to keep one half of cores undisturbed for long-term preservation.

Skeletal Chemistry (Raman)

*Sample Collection:* Raman spectroscopy can be applied to skeletal cores or coral fragments. Coral fragments are initially soaked in ~3% sodium hypochlorite (bleach) for at least 1 hour, or until white (bleach can be replaced if necessary) to remove tissue.

*Short-Term Preservation of Samples*: Samples for Raman analysis should ideally never be stored in any chemical preservative or in water. Since Raman analyses are conducted on skeletal surfaces at micron-scales, any minor dissolution or precipitation of new aragonite crystals has the potential to influence the Raman data.

*Sample Processing:* Heating of skeleton samples prior to Raman analysis can substantially reduce the data quality (DeCarlo, 2018). This is because heating causes “annealing” of samples, a process in which disorder in the crystal structure of the skeleton is alleviated. Since Raman analyses depend on characterizing skeletal disorder, heating can thus change the Raman data in such a way that comparisons among samples may not be meaningful. The sensitivity of Raman analyses to heated coral skeletons is poorly known, though. Heating to 60℃ for minutes to several hours to dry skeletal powders does not seem to have a substantial effect, whereas heating to 140℃ for 16 hours dramatically changes the resulting Raman spectra (DeCarlo, 2018), but heat procedures in between these two have not yet been tested.

*Long-Term Preservation of Processed Samples*: Skeletal material can be stored at room temperature indefinitely if stored in a sealed receptacle.

*Archiving of Unprocessed Samples*: Bleached coral skeletons which were not ground may be stored indefinitely at room temperature.

Symbiodiniaceae Density and Mitotic Index

*Sample Collection:* Samples for Symbiodiniaceae quantification are typically collected as whole coral fragments, but planulae and gametes are acceptable and generally require 1cm^2^ of coral tissue, although more may be required if corals are significantly bleached. Samples are best kept cold on ice or at 4℃. While rapid freezing and short-term storage with dry ice is acceptable, one should ensure that cell breakage is not occurring by freezing, or that the percent loss in Symbiodiniaceae cells due to freezing is consistent and quantifiable.

*Short-Term Preservation of Samples:* Sample storage in an ultra-cold freezer is commonly employed for short and long-term storage. Samples can also be kept short-term at −20℃ or 4℃, although not optimal. If samples are chemically preserved (e.g., by glutaraldehyde or formaldehyde fixation) storage at 4℃ is acceptable.

*Sample Processing:* Tissues must be homogenized prior to counting symbionts and is typically done using filtered seawater (natural or artificial). Tissue homogenization can be done on airbrushed/water-piked tissues using a glass or electronic homogenizer, or by grinding whole fragments with mortar and pestle. Hemocytometers, flow cytometry, and other automated cell counter methods benefit from separation of symbiont cells from host tissue/mucus/skeleton via differential centrifugation prior to counting to avoid clogging the instrument (Krediet et al., 2015; McLachlan, Juracka & Grottoli, 2020). Fixation can aid in long-term storage prior to counting but can affect cell counts. Freeze/thaw cycles can lyse symbiont cells and depress cell counts (Krediet et al., 2015) and repeat freeze-thaw cycles should be avoided. A study of the effects of freezing and fixation on the Symbiodiniaceae density of anemones found that the order in which samples are frozen/fixed and homogenized is important, and can alter the density of cells which are subsequently counted (Krediet et al., 2015).

*Long-Term Preservation of Processed Samples:* Methods for symbiont counts are destructive and no processed sample remains.

*Archiving of Unprocessed Samples:* Remaining fragments, planulae, gametes, and/or tissue homogenates can be stored long-term at −80℃, but, as noted above, freeze/thaw cycles can lyse symbiont cells and repeat cycles should be avoided.

Coral Color Analysis from Digital Imagery

*Collection of Digital Images:* Coral color analysis is conducted using digital images taken of a live coral colony either *in situ* or *ex-situ* in a photo studio (Amid et al., 2018). Photographs must be taken prior to any preservation or processing of tissue, such as freezing, use of preservatives or fixatives, airbrushing etc., to ensure no alteration of the original coral color occurs. Depending on the method of image analyses intended (e.g., the Red Green Blue Color Model (Edmunds, Gates & Gleason, 2003; Siebeck et al., 2006; Winters et al., 2009; Voolstra et al., 2020), or the Greyscale Model (Chow et al., 2016; Amid et al., 2018), it may be necessary to photograph the coral colony next to a black, white, or color standard reference card. It is essential that the coral colony and the reference card receive the same uniform illumination/light field. If possible, image coral colonies from multiple angles in order to get a good representation of bleaching degree and color (McLachlan & Grottoli, 2021). Capturing images using the camera’s raw settings will avoid loss of information during the image compression compared to other photo formats (e.g., JPEG).

*Processing of Digital Images*: Depending on the method of image analysis used, digital image corrections may be necessary, for example external light normalization (Winters et al., 2009) or conversion of digital images to 8-bit grey scale (Chow et al., 2016; Amid et al., 2018) using an image analysis software such as ImageJ (Rasband, W.S., 1997) or Adobe Photoshop (*Adobe Photoshop CS*, 2004).

*Preservation of Digital Images*: Digital images may be stored indefinitely via cloud or physical storage.

Measuring Coral Size

*Data Collection:* Coral specimen size is a basic but essential measurement that is often necessary as the denominator used to standardize the quantity of another measured variable to adjust for the amount of coral analyzed. Researchers can inflate the uncertainty of their measured variables of interest if there is considerable measurement error in what is used to normalize the sample measurement. To avoid error inflation, there are several ways to measure coral size (Supplementary Material Table 1), and many of these can be used as an integrated measure of coral “health” if measurements are taken at two time points (e.g.., measuring growth, see appendix in (Grottoli et al., 2021).

**Supplemental Table 1.**Measures of coral specimen size/growth are essential metadata when normalized to other variables (e.g., symbiont density, calcification, etc.). Measures of size/growth over time can also be experimental response variables if measured through time (e.g., before and after a treatment is applied, before and after a natural bleaching event etc.). This table summarizes possible measures of size/growth and their utility for normalization. Methods which are *italicized* are categorized as invasive, impacting the coral colony through substantial contact or destruction.

| **Measure of size/growth** | **Example unit** | **For Normalization** | **Economical method** | **Resource-intensive method** |
| --- | --- | --- | --- | --- |
| Length/height/width/ diameter | cm | Not ideal | Direct measurement with tape or calipers | NA |
| Contoured surface area | cm^2^ | Highly desirable | *Wax* *dipped (Stimson & Kinzie, 1991)*, *foil* *wrapped (Marsh, 1970)*, *latex* *dipped (Meyer & Schultz, 1985)*, *geometric shape fitting* (Odum, 1995; Jones et al., 2008), *spectrophotometry* *using* *dye* (Hoegh-Guldberg, 1988)*,* | Photogrammetry (Bythell, Pan & Lee, 2001; Courtney et al., 2007; Lavy et al., 2015), X-ray computed tomography (Laforsch et al., 2008; Naumann et al., 2009), handheld laser scanning (Holmes, 2008), 3D laser scanning (Enochs et al., 2014), stereo video (Cocito et al., 2003) |
| Planar area footprint | cm^2^ | Highly desirable | Direct measurement with tape or calipers (Kuffner, Hickey & Morrison, 2013) | From photographs (Rahav et al., 1991; Edmunds & Elahi, 2007; Madin et al., 2014; Neal et al., 2015; Kuffner et al., 2019) |
| Volume | cm^3^ | Not ideal | Calipers or tape | Photogrammetry (Lavy et al., 2015) |
| Linear extension rate | cm yr^-1^ | Highly desirable | *Tagging* (Shinn, 1966), *staining with alizarin/ alizarin complexone /calcein/oxytetracycline* (Barnes, Beck & Schultz, 1970; Holcomb, Cohen & McCorkle, 2013)*,* time lapse photography (Barnes & Crossland, 1980), direct measurement with tape (Miller, Weil & Szmant, 2000) or calipers (Stimson, 1985) | *X-radiography* (Lough & Barnes, 2000)*, luminescent lines* (D’Olivo, McCulloch & Judd, 2013; Tanzil et al., 2013)*, stable isotope profiling* (Felis, Pätzold & Loya, 2003; Storz & Gischler, 2011), *in situ* laser measurements (Vago, Gill & Collingwood, 1997), |
| Bulk density | g cm^-3^ | Not commonly used | Wet weight of skeleton divided by height (Atkinson, Carlson & Crow, 1995) | *X-ray densitometry* (Buddemeier, 1974)*, gamma densitometry* (Chalker & Barnes, 1990)*, Computerized tomography densitometry* (Logan & Anderson, 1991) |
| Calcification rate  *NB: can be calculated as the product of extension and density* | g CaCO_3_ cm^-2^ y^-1^ | Highly desirable | *Buoyant weight* (Jokiel, Maragos & Franzisket, 1978; Davies, 1989)*, in situ* buoyant weight (Bak, 1973; Herler & Dirnwöber, 2011) | *Radioisotope incorporation* (Goreau, 1959; Tambutté et al., 1995)*,* alkalinity anomaly (Smith, 1973) |

Literature Cited:

*Adobe Photoshop CS*. 2004. Berkeley, CA: Peachpit Press.

Ainsworth TD, Krause L, Bridge T, Torda G, Raina J-B, Zakrzewski M, Gates RD, Padilla-Gamiño JL, Spalding HL, Smith C, Woolsey ES, Bourne DG, Bongaerts P, Hoegh-Guldberg O, Leggat W. 2015. The coral core microbiome identifies rare bacterial taxa as ubiquitous endosymbionts. *The International Society for Microbial Ecology Journal* 9:2261–2274. DOI: 10.1038/ismej.2015.39.

Alamri A, Nam JY, Blancato JK. 2017. Fluorescence In Situ Hybridization of Cells, Chromosomes, and Formalin-Fixed Paraffin-Embedded Tissues. In: Espina V ed. *Molecular Profiling: Methods and Protocols*. Methods in Molecular Biology. New York, NY: Springer, 265–279. DOI: 10.1007/978-1-4939-6990-6_17.

Allis CD, Caparros M-L, Jenuwein T, Reinberg D. 2015. *Epigenetics*. Cold Spring Harbor, New York: CSH Press, Cold Spring Harbor Laboratory Press.

Alpert AE, Cohen AL, Oppo DW, DeCarlo TM, Gaetani GA, Hernandez-Delgado EA, Winter A, Gonneea ME. 2017. Twentieth century warming of the tropical Atlantic captured by Sr-U paleothermometry. *Paleoceanography* 32:146–160. DOI: 10.1002/2016PA002976.

Amid C, Olstedt M, Gunnarsson JS, Le Lan H, Tran Thi Minh H, Van den Brink PJ, Hellström M, Tedengren M. 2018. Additive effects of the herbicide glyphosate and elevated temperature on the branched coral Acropora formosa in Nha Trang, Vietnam. *Environmental Science and Pollution Research* 25:13360–13372. DOI: 10.1007/s11356-016-8320-7.

Atkinson MJ, Carlson B, Crow GL. 1995. Coral growth in high-nutrient, low-pH seawater: a case study of corals cultured at the Waikiki Aquarium, Honolulu, Hawaii. *Coral Reefs* 14:215–223. DOI: 10.1007/BF00334344.

Bahamonde PA, Feswick A, Isaacs MA, Munkittrick KR, Martyniuk CJ. 2016. Defining the role of omics in assessing ecosystem health: Perspectives from the Canadian environmental monitoring program. *Environmental Toxicology and Chemistry* 35:20–35. DOI: 10.1002/etc.3218.

Bak RPM. 1973. Coral weight increment in situ. A new method to determine coral growth. *Marine Biology* 20:45–49. DOI: 10.1007/BF00387673.

Barnes D, Beck RE, Schultz JS. 1970. Coral Skeletons : An Explanation of Their Growth and Structure Author ( s ): David J . Barnes Published by : American Association for the Advancement of Science Stable URL : http://www.jstor.org/stable/1730509 REFERENCES Linked references are available on. 170:1305–1308.

Barnes DJ, Crossland CJ. 1980. Diurnal and seasonal variations in the growth of a staghorn coral measured by time‐lapse photography. *Limnology and Oceanography* 25:1113–1117. DOI: 10.4319/lo.1980.25.6.1113.

Baumann J, Grottoli AG, Hughes AD, Matsui Y. 2014. Photoautotrophic and heterotrophic carbon in bleached and non-bleached coral lipid acquisition and storage. *Journal of Experimental Marine Biology and Ecology* 461:469–478. DOI: 10.1016/j.jembe.2014.09.017.

Boiseau M, Juillet-Leclerc A, Yiou P, Salvat B, Isdale P, Guillaume M. 1998. Atmospheric and oceanic evidences of El Niño-Southern Oscillation events in the south central Pacific Ocean from coral stable isotopic records over the last 137 years. *Paleoceanography* 13:671–685. DOI: 10.1029/98PA02502.

Brown BE, Le Tissier MDA, Bythell JC. 1995. Mechanisms of bleaching deduced from histological studies of reef corals sampled during a natural bleaching event. *Marine Biology* 122:655–663. DOI: 10.1007/BF00350687.

Buddemeier RW. 1974. Environmental controls over annual and lunar monthly cycles in hermatypic coral calcification. *Proceedings of the 2nd International Coral Reef Symposium.* 2:259–267.

Bythell JC, Barer MR, Cooney RP, Guest JR, O’Donnell AG, Pantos O, Tissier MDAL. 2002. Histopathological methods for the investigation of microbial communities associated with disease lesions in reef corals. *Letters in Applied Microbiology* 34:359–364. DOI: 10.1046/j.1472-765X.2002.01097.x.

Bythell J, Pan P, Lee J. 2001. Three-dimensional morphometric measurements of reef corals using underwater photogrammetry techniques. *Coral Reefs* 20:193–199. DOI: 10.1007/s003380100157.

Chalker BE, Barnes DJ. 1990. Gamma densitometry for the measurement of skeletal density. *Coral Reefs* 9:11–23. DOI: 10.1007/BF00686717.

Cheng Y-S, Zheng Y, VanderGheynst JS. 2011. Rapid Quantitative Analysis of Lipids Using a Colorimetric Method in a Microplate Format. *Lipids* 46:95–103. DOI: 10.1007/s11745-010-3494-0.

Chow MH, Tsang RHL, Lam EKY, Ang P. 2016. Quantifying the degree of coral bleaching using digital photographic technique. *Journal of Experimental Marine Biology and Ecology* 479:60–68. DOI: 10.1016/j.jembe.2016.03.003.

Christie WW. 2003. Lipid analysis: isolation, separation, identification and structural analysis of lipids.

Christie WW, Han X. 2010. *Lipid Analysis: Isolation, Separation, Identification and Lipidomic Analysis: Fourth Edition*. Elsevier Ltd. DOI: 10.1533/9780857097866.

Cocito S, Sgorbini S, Peirano A, Valle M. 2003. 3-D reconstruction of biological objects using underwater video technique and image processing. *Journal of Experimental Marine Biology and Ecology* 297:57–70. DOI: 10.1016/S0022-0981(03)00369-1.

Conlan JA, Rocker MM, Francis DS. 2017. A comparison of two common sample preparation techniques for lipid and fatty acid analysis in three different coral morphotypes reveals quantitative and qualitative differences. *PeerJ* 5:e3645. DOI: 10.7717/peerj.3645.

Conti-Jerpe IE, Thompson PD, Wong CWM, Oliveira NL, Duprey NN, Moynihan MA, Baker DM. 2020. Trophic strategy and bleaching resistance in reef-building corals. *Science Advances* 6:eaaz5443. DOI: 10.1126/sciadv.aaz5443.

Correa AMS, Ainsworth TD, Rosales SM, Thurber AR, Butler CR, Vega Thurber RL. 2016. Viral Outbreak in Corals Associated with an In Situ Bleaching Event: Atypical Herpes-Like Viruses and a New Megavirus Infecting Symbiodinium. *Frontiers in Microbiology* 7. DOI: 10.3389/fmicb.2016.00127.

Correa AMS, Brandt ME, Smith TB, Thornhill DJ, Baker AC. 2009. Symbiodinium associations with diseased and healthy scleractinian corals. *Coral Reefs* 28:437–448. DOI: 10.1007/s00338-008-0464-6.

Corredor JE, Bruckner AW, Muszynski FZ, Armstrong RA, García R, Morell JM. 2000. UV-absorbing compounds in three species of Caribbean zooxanthellate corals: Depth distribution and spectral response. *Bulletin of Marine Science* 67:821–830.

Courtney LA, Fisher WS, Raimondo S, Oliver LM, Davis WP. 2007. Estimating 3-dimensional colony surface area of field corals. *Journal of Experimental Marine Biology and Ecology* 351:234–242. DOI: 10.1016/j.jembe.2007.06.021.

Davies SP. 1989. Short-term growth measurements of corals using an accurate buoyant weighing technique. *Marine Biology* 101:389–395. DOI: 10.1007/BF00428135.

Dawson MN, Raskoff KA, Jacobs DK. 1998. Field preservation of marine invertebrate tissue for DNA analyses. *Molecular Marine Biology and Biotechnology* 7:145–152.

DeCarlo TM. 2018. Characterizing coral skeleton mineralogy with Raman spectroscopy. *Nature Communications* 9:5325. DOI: 10.1038/s41467-018-07601-3.

Dimond JL, Roberts SB. 2020. Convergence of DNA Methylation Profiles of the Reef Coral Porites astreoides in a Novel Environment. *Frontiers in Marine Science* 6:792. DOI: 10.3389/fmars.2019.00792.

D’Olivo JP, McCulloch MT. 2017. Response of coral calcification and calcifying fluid composition to thermally induced bleaching stress. *Scientific Reports* 7:2207. DOI: 10.1038/s41598-017-02306-x.

D’Olivo JP, McCulloch MT, Judd K. 2013. Long-term records of coral calcification across the central Great Barrier Reef: Assessing the impacts of river runoff and climate change. *Coral Reefs* 32:999–1012. DOI: 10.1007/s00338-013-1071-8.

DuBois Michel, Gilles KA, Hamilton JK, Rebers PA, Smith Fred. 1956. Colorimetric Method for Determination of Sugars and Related Substances. *Analytical Chemistry* 28:350–356. DOI: 10.1021/ac60111a017.

Edmunds PJ, Elahi R. 2007. The demographics of a 15-year decline in cover of the Caribbean reef coral Montastraea annularis. *Ecological Monographs* 77:3–18. DOI: 10.1890/05-1081.

Edmunds P, Gates R. 2002. Normalizing physiological data for scleractinian corals. *Coral Reefs* 21:193–197. DOI: 10.1007/s00338-002-0214-0.

Edmunds PJ, Gates RD, Gleason DF. 2003. The tissue composition of Montastraea franksi during a natural bleaching event in the Florida Keys. *Coral Reefs* 22:54–62. DOI: 10.1007/s00338-003-0278-5.

Ellis B, Grant K, Mallela J, Abram N. 2019. Is XRF core scanning a viable method for coral palaeoclimate temperature reconstructions? *Quaternary International* 514:97–107. DOI: 10.1016/j.quaint.2018.11.044.

Enochs IC, Manzello DP, Carlton R, Schopmeyer S, van Hooidonk R, Lirman D. 2014. Effects of light and elevated pCO2 on the growth and photochemical efficiency of Acropora cervicornis. *Coral Reefs* 33:477–485. DOI: 10.1007/s00338-014-1132-7.

Ezzat L, Fine M, Maguer J-F, Grover R, Ferrier-Pagès C. 2017. Carbon and Nitrogen Acquisition in Shallow and Deep Holobionts of the Scleractinian Coral S. pistillata. *Frontiers in Marine Science* 4:102. DOI: 10.3389/fmars.2017.00102.

Felis T. 2020. Extending the Instrumental Record of Ocean-Atmosphere Variability into the Last Interglacial Using Tropical Corals. *Oceanography* 33:68–79.

Felis T, Pätzold J, Loya Y. 2003. Mean oxygen-isotope signatures in Porites spp. corals: Inter-colony variability and correction for extension-rate effects. *Coral Reefs* 22:328–336. DOI: 10.1007/s00338-003-0324-3.

Felis T, Pätzold J, Loya Y, Wefer G. 1998. Vertical water mass mixing and plankton blooms recorded in skeletal stable carbon isotopes of a Red Sea coral. *Journal of Geophysical Research: Oceans* 103:30731–30739. DOI: 10.1029/98JC02711.

Felis T, Suzuki A, Kuhnert H, Dima M, Lohmann G, Kawahata H. 2009. Subtropical coral reveals abrupt early-twentieth-century freshening in the western North Pacific Ocean. *Geology* 37:527–530. DOI: 10.1130/G25581A.1.

Ferrier-Pagès C, Martinez S, Grover R, Cybulski J, Shemesh E, Tchernov D. 2021. Tracing the Trophic Plasticity of the Coral–Dinoflagellate Symbiosis Using Amino Acid Compound-Specific Stable Isotope Analysis. *Microorganisms* 9:182. DOI: 10.3390/microorganisms9010182.

Fiehn O, Robertson D, Griffin J, van der Werf M, Nikolau B, Morrison N, Sumner LW, Goodacre R, Hardy NW, Taylor C, Fostel J, Kristal B, Kaddurah-Daouk R, Mendes P, van Ommen B, Lindon JC, Sansone S-A. 2007. The metabolomics standards initiative (MSI). *Metabolomics* 3:175–178. DOI: 10.1007/s11306-007-0070-6.

Gaither MR, Szabó Z, Crepeau MW, Bird CE, Toonen RJ. 2011. Preservation of corals in salt-saturated DMSO buffer is superior to ethanol for PCR experiments. *Coral Reefs* 30:329–333. DOI: 10.1007/s00338-010-0687-1.

Gierz S, Ainsworth TD, Leggat W, Gierz S, Ainsworth TD, Leggat W. 2020. Diverse symbiont bleaching responses are evident from 2-degree heating week bleaching conditions as thermal stress intensifies in coral. *Marine and Freshwater Research* 71:1149–1160. DOI: 10.1071/MF19220.

Giry C, Felis T, Kölling M, Scheffers S. 2010. Geochemistry and skeletal structure of Diploria strigosa, implications for coral-based climate reconstruction. *Palaeogeography, Palaeoclimatology, Palaeoecology* 298:378–387. DOI: 10.1016/j.palaeo.2010.10.022.

Goreau TJ. 1959. The physiology of skeleton formation in corals. I. A method for measuring the rate of calcium deposition by corals under different conditions. *Biological Bulletin* 116:59–75.

Gray MA, Pratte ZA, Kellogg CA. 2013. Comparison of DNA preservation methods for environmental bacterial community samples. *FEMS Microbiology Ecology* 83:468–477. DOI: 10.1111/1574-6941.12008.

Greene A, Leggat W, Donahue MJ, Raymundo LJ, Caldwell JM, Moriarty T, Heron SF, Ainsworth TD. 2020. Complementary sampling methods for coral histology, metabolomics and microbiome. *Methods in Ecology and Evolution* 11:1012–1020. DOI: 10.1111/2041-210X.13431.

Grottoli AG. 2002. Effect of light and brine shrimp on skeletal δ13C in the Hawaiian coral Porites compressa: a tank experiment. *Geochimica et Cosmochimica Acta* 66:1955–1967. DOI: 10.1016/S0016-7037(01)00901-2.

Grottoli AG, Eakin CM. 2007. A review of modern coral δ18O and Δ14C proxy records. *Earth-Science Reviews* 81:67–91. DOI: 10.1016/j.earscirev.2006.10.001.

Grottoli AG, Rodrigues LJ, Matthews KA, Palardy JE, Gibb OT. 2005. Pre-treatment effects on coral skeletal δ13C and δ18O. *Chemical Geology* 221:225–242. DOI: 10.1016/j.chemgeo.2005.05.004.

Grottoli AG, Tchernov D, Winters G. 2017. Physiological and Biogeochemical Responses of Super-Corals to Thermal Stress from the Northern Gulf of Aqaba, Red Sea. *Frontiers in Marine Science* 0. DOI: 10.3389/fmars.2017.00215.

Grottoli AG, Toonen RJ, Woesik R van, Thurber RV, Warner ME, McLachlan RH, Price JT, Bahr KD, Baums IB, Castillo KD, Coffroth MA, Cunning R, Dobson KL, Donahue MJ, Hench JL, Iglesias-Prieto R, Kemp DW, Kenkel CD, Kline DI, Kuffner IB, Matthews JL, Mayfield AB, Padilla-Gamiño JL, Palumbi S, Voolstra CR, Weis VM, Wu HC. 2021. Increasing comparability among coral bleaching experiments. *Ecological Applications* 31:e02262. DOI: 10.1002/eap.2262.

Grottoli AG, Wellington GM. 1999. Effect of light and zooplankton on skeletal δ^13^C values in the eastern Pacific corals Pavona clavus and Pavona gigantea. *Coral Reefs* 18.

Hara A, Radin NS. 1978. Lipid extraction of tissues with a low-toxicity solvent. *Analytical Biochemistry* 90:420–426. DOI: 10.1016/0003-2697(78)90046-5.

Harii S, Yasuda N, Rodriguez-Lanetty M, Irie T, Hidaka M. 2009. Onset of symbiosis and distribution patterns of symbiotic dinoflagellates in the larvae of scleractinian corals. *Marine Biology* 156:1203–1212. DOI: 10.1007/s00227-009-1162-9.

Harland AD, Fixter LM, Davies PS, Anderson RA. 1991. Distribution of lipids between the zooxanthellae and animal compartment in the symbiotic sea anemoneAnemonia viridis: Wax esters, triglycerides and fatty acids. *Marine Biology* 110:13–19. DOI: 10.1007/BF01313087.

Hathorne EC, Felis T, James RH, Thomas A. 2011. Laser ablation ICP-MS screening of corals for diagenetically affected areas applied to Tahiti corals from the last deglaciation. *Geochimica et Cosmochimica Acta* 75:1490–1506. DOI: 10.1016/j.gca.2010.12.011.

Hathorne EC, Felis T, Suzuki A, Kawahata H, Cabioch G. 2013. Lithium in the aragonite skeletons of massive Porites corals: A new tool to reconstruct tropical sea surface temperatures. *Paleoceanography* 28:143–152. DOI: 10.1029/2012PA002311.

Hayes RL, Bush PG. 1990. Microscopic observations of recovery in the reef-building scleractinian coral, Montastrea annularis, after bleaching on a Cayman reef. *Coral Reefs* 8:203–209. DOI: 10.1007/BF00265012.

Hemming NG, Hanson GN. 1992. Boron isotopic composition and concentration in modern marine carbonates. *Geochimica et Cosmochimica Acta* 56:537–543. DOI: 10.1016/0016-7037(92)90151-8.

Hemond EM, Vollmer SV. 2015. Diurnal and nocturnal transcriptomic variation in the Caribbean staghorn coral, Acropora cervicornis. *Molecular Ecology* 24:4460–4473. DOI: 10.1111/mec.13320.

Herler J, Dirnwöber M. 2011. A simple technique for measuring buoyant weight increment of entire, transplanted coral colonies in the field. *Journal of Experimental Marine Biology and Ecology* 407:250–255. DOI: 10.1016/j.jembe.2011.06.022.

Hernandez-Agreda A, Leggat W, Ainsworth TD. 2018. A Comparative Analysis of Microbial DNA Preparation Methods for Use With Massive and Branching Coral Growth Forms. *Frontiers in Microbiology* 0. DOI: 10.3389/fmicb.2018.02146.

Hillyer KE, Dias DA, Lutz A, Roessner U, Davy SK. 2017. Mapping carbon fate during bleaching in a model cnidarian symbiosis: the application of 13C metabolomics. *New Phytologist* 214:1551–1562. DOI: 10.1111/nph.14515.

Hillyer KE, Dias D, Lutz A, Roessner U, Davy SK. 2018. 13C metabolomics reveals widespread change in carbon fate during coral bleaching. *Metabolomics* 14:12. DOI: 10.1007/s11306-017-1306-8.

Hoadley KD, Lewis AM, Wham DC, Pettay DT, Grasso C, Smith R, Kemp DW, LaJeunesse TC, Warner ME. 2019. Host–symbiont combinations dictate the photo-physiological response of reef-building corals to thermal stress. *Scientific Reports* 9:9985. DOI: 10.1038/s41598-019-46412-4.

Hoegh-Guldberg O. 1988. A method for determining the surface area of corals. *Coral Reefs* 7:113–116. DOI: 10.1007/BF00300970.

Holcomb M, Cohen AL, McCorkle DC. 2013. An evaluation of staining techniques for marking daily growth in scleractinian corals. *Journal of Experimental Marine Biology and Ecology* 440:126–131. DOI: 10.1016/j.jembe.2012.12.003.

Holmes G. 2008. Estimating three-dimensional surface areas on coral reefs. *Journal of Experimental Marine Biology and Ecology* 365:67–73. DOI: 10.1016/j.jembe.2008.07.045.

Holm-Hansen O, Riemann B. 1978. Chlorophyll a Determination: Improvements in Methodology. *Oikos* 30:438–447. DOI: 10.2307/3543338.

Hönisch B, Hemming NG, Grottoli AG, Amat A, Hanson GN, Bijma J. 2004. Assessing scleractinian corals as recorders for paleo-pH: Empirical calibration and vital effects. *Geochimica et Cosmochimica Acta* 68:3675–3685. DOI: 10.1016/j.gca.2004.03.002.

Hooker, SB. 2005. *Second SeaWiFS HPLC Analysis Round-robin Experiment (SeaHARRE-2)*. National Aeronautics and Space Administration, Goddard Space Flight Center.

Hughes AD, Grottoli AG. 2013. Heterotrophic Compensation: A Possible Mechanism for Resilience of Coral Reefs to Global Warming or a Sign of Prolonged Stress? *PLOS ONE* 8:e81172. DOI: 10.1371/journal.pone.0081172.

Hughes AD, Grottoli AG, Pease TK, Matsui Y. 2010. Acquisition and assimilation of carbon in non-bleached and bleached corals. *Marine Ecology Progress Series* 420:91–101. DOI: 10.3354/meps08866.

Jeffrey SW, Humphrey GF. 1975. New spectrophotometric equations for determining chlorophylls a, b, c1 and c2 in higher plants, algae and natural phytoplankton. *Biochemie und Physiologie der Pflanzen* 167:191–194. DOI: 10.1016/S0015-3796(17)30778-3.

Ji X, Wang M, Li L, Chen F, Zhang Y, Li Q, Zhou J. 2017. The Impact of Repeated Freeze–Thaw Cycles on the Quality of Biomolecules in Four Different Tissues. *Biopreservation and Biobanking* 15:475–483. DOI: 10.1089/bio.2017.0064.

Jokiel PL, Maragos JE, Franzisket L. 1978. Coral growth: buoyant weight technique. In: *Coral Reefs: Research Methods*.

Jones AM, Cantin NE, Berkelmans R, Sinclair B, Negri AP. 2008. A 3D modeling method to calculate the surface areas of coral branches. *Coral Reefs* 27:521–526. DOI: 10.1007/s00338-008-0354-y.

Kemp DW, Fitt WK, Schmidt GW. 2008. A microsampling method for genotyping coral symbionts. *Coral Reefs* 27:289–293. DOI: 10.1007/s00338-007-0333-8.

Krediet CJ, DeNofrio JC, Caruso C, Burriesci MS, Cella K, Pringle JR. 2015. Rapid, Precise, and Accurate Counts of Symbiodinium Cells Using the Guava Flow Cytometer, and a Comparison to Other Methods. *PLOS ONE* 10:e0135725. DOI: 10.1371/journal.pone.0135725.

Kuffner IB, Hickey TD, Morrison JM. 2013. Calcification rates of the massive coral Siderastrea siderea and crustose coralline algae along the Florida Keys (USA) outer-reef tract. *Coral Reefs* 32:987–997. DOI: 10.1007/s00338-013-1047-8.

Kuffner IB, Toth LT, Hudson JH, Goodwin WB, Stathakopoulos A, Bartlett LA, Whitcher EM. 2019. Improving estimates of coral reef construction and erosion with in situ measurements. *Limnology and Oceanography* 64:2283–2294. DOI: 10.1002/lno.11184.

Laforsch C, Christoph E, Glaser C, Naumann MS, Wild C, Niggl W. 2008. A precise and non-destructive method to calculate the surface area in living scleractinian corals using X-ray computed tomography and 3D modeling. *Coral Reefs* 27:811–820. DOI: 10.1007/s00338-008-0405-4.

LaVigne M, Grottoli AG, Palardy JE, Sherrell RM. 2016. Multi-colony calibrations of coral Ba/Ca with a contemporaneous in situ seawater barium record. *Geochimica et Cosmochimica Acta* 179:203–216. DOI: 10.1016/j.gca.2015.12.038.

Lavy A, Eyal G, Neal B, Keren R, Loya Y, Ilan M. 2015. A quick, easy and non-intrusive method for underwater volume and surface area evaluation of benthic organisms by 3D computer modelling. *Methods in Ecology and Evolution* 6:521–531. DOI: 10.1111/2041-210X.12331.

van Leeuwe MA, Villerius LA, Roggeveld J, Visser RJW, Stefels J. 2006. An optimized method for automated analysis of algal pigments by HPLC. *Marine Chemistry* 102:267–275. DOI: 10.1016/j.marchem.2006.05.003.

Levy O, Appelbaum L, Leggat W, Gothlif Y, Hayward DC, Miller DJ, Hoegh-Guldberg O. 2007. Light-Responsive Cryptochromes from a Simple Multicellular Animal, the Coral Acropora millepora. *Science* 318:467–470. DOI: 10.1126/science.1145432.

Levy O, Dubinsky Z, Achituv Y. 2003. Photobehavior of stony corals: responses to light spectra and intensity. *Journal of Experimental Biology* 206:4041–4049. DOI: 10.1242/jeb.00622.

Liu Y, Li X, Zeng Z, Yu H-M, Huang F, Felis T, Shen C-C. 2019. Annually-resolved coral skeletal δ138/134Ba records: A new proxy for oceanic Ba cycling. *Geochimica et Cosmochimica Acta* 247:27–39. DOI: 10.1016/j.gca.2018.12.022.

Logan A, Anderson H. 1991. Skeletal extension growth rate assessment in corals, using CT scan imagery. *Bulletin of Marine Science* 49:847–850.

Lough JM, Barnes DJ. 2000. Environmental controls on growth of the massive coral Porites. *Journal of Experimental Marine Biology and Ecology* 245:225–243. DOI: 10.1016/S0022-0981(99)00168-9.

Madin JS, Baird AH, Dornelas M, Connolly SR. 2014. Mechanical vulnerability explains size-dependent mortality of reef corals. *Ecology Letters* 17:1008–1015. DOI: 10.1111/ele.12306.

Marsh JA. 1970. Primary productivity of reef-building calcareous red algae. *Ecology* 51:255–263. DOI: 10.1038/news050808-1.

Masuko T, Minami A, Iwasaki N, Majima T, Nishimura S-I, Lee YC. 2005. Carbohydrate analysis by a phenol–sulfuric acid method in microplate format. *Analytical Biochemistry* 339:69–72. DOI: 10.1016/j.ab.2004.12.001.

Matthews JL, Cunning R, Witson-Williams R, Oakley CA, Lutz A, Roessner U, Grossman AR, Weis VM, Gates RD, Davy SK. 2022. Chapter 15 - The metabolic significance of symbiont community composition in the coral-algal symbiosis☆. In: Beale DJ, Hillyer KE, Warden AC, Jones OAH eds. *Applied Environmental Metabolomics*. Academic Press, 211–229. DOI: 10.1016/B978-0-12-816460-0.00016-2.

Matthews KA, McDonough WF, Grottoli AG. 2006. Cadmium measurements in coral skeleton using isotope dilution–inductively coupled plasma–mass spectrometry. *Geochemistry, Geophysics, Geosystems* 7. DOI: 10.1029/2006GC001352.

McClanahan TR, McLaughlin SM, Davy JE, Wilson WH, Peters EC, Price KL, Maina J. 2004. Observations of a new source of coral mortality along the Kenyan coast. *Hydrobiologia* 530:469–479. DOI: 10.1007/s10750-004-2672-6.

McCulloch MT, D’Olivo JP, Falter J, Holcomb M, Trotter JA. 2017. Coral calcification in a changing World and the interactive dynamics of pH and DIC upregulation. *Nature Communications* 8:15686. DOI: 10.1038/ncomms15686.

McLachlan R, Dobson K, Grottoli AG. 2020. Quantification of Total Biomass in Ground Coral Samples. DOI: 10.17504/protocols.io.bdyai7se.

McLachlan R, Grottoli A. 2021.Image Analysis to Quantify Coral Bleaching Using Greyscale Model. *Available at* *https://www.protocols.io/view/image-analysis-to-quantify-coral-bleaching-using-g-bx8wprxe* (accessed November 18, 2021).

McLachlan R, Juracka C, Grottoli AG. 2020. Symbiodiniaceae Enumeration in Ground Coral Samples Using Countess^TM^ II FL Automated Cell Counter. DOI: 10.17504/protocols.io.bdc5i2y6.

McLachlan R, Munoz-Garcia A, Grottoli AG. 2020. Extraction of Total Soluble Lipid from Ground Coral Samples. DOI: 10.17504/protocols.io.bc4qiyvw.

McLachlan R, Price, Jamie, Dobson, Kerri, Weisleder, Noah, Grottoli, Andrea G. 2020. Microplate Assay for Quantification of Soluble Protein in Ground Coral Samples. DOI: 10.17504/protocols.io.bdc8i2zw.

Meyer JL, Schultz ET. 1985. Tissue condition and growth rate of corals associated with schooling fish. *Limnology and Oceanography* 30:157–166. DOI: 10.4319/lo.1985.30.1.0157.

Miller MW, Weil E, Szmant AM. 2000. Coral recruitment and juvenile mortality as structuring factors for reef benthic communities in Biscayne National Park, USA. *Coral Reefs* 19:115–123. DOI: 10.1007/s003380000079.

Muscatine L, Porter JW, Kaplan IR. 1989. Resource partitioning by reef corals as determined from stable isotope composition. *Marine Biology* 100:185–193. DOI: 10.1007/BF00391957.

Mushtaq MY, Choi YH, Verpoorte R, Wilson EG. 2014. Extraction for Metabolomics: Access to The Metabolome. *Phytochemical Analysis* 25:291–306. DOI: 10.1002/pca.2505.

Naumann MS, Niggl W, Laforsch C, Glaser C, Wild C. 2009. Coral surface area quantification-evaluation of established techniques by comparison with computer tomography. *Coral Reefs* 28:109–117. DOI: 10.1007/s00338-008-0459-3.

Neal BP, Lin TH, Winter RN, Treibitz T, Beijbom O, Kriegman D, Kline DI, Greg Mitchell B. 2015. Methods and measurement variance for field estimations of coral colony planar area using underwater photographs and semi-automated image segmentation. *Environmental Monitoring and Assessment* 187. DOI: 10.1007/s10661-015-4690-4.

Odum HTO& EP. 1995. Trophic Structure and Productivity of a Windward Coral Reef Community on Eniwetok Atoll Author ( s ): Howard T . Odum and Eugene P . Odum Published by : Ecological Society of America Stable URL : http://www.jstor.org/stable/1943285 . Your use of the JSTOR. *Ecological Monograph* 25:291–320.

Piniak GA, Lipschultz F, McClelland J. 2003. Assimilation and partitioning of prey nitrogen within two anthozoans and their endosymbiotic zooxanthellae. *Marine Ecology Progress Series* 262:125–136. DOI: 10.3354/meps262125.

Porra RJ, Thompson WA, Kriedemann PE. 1989. Determination of accurate extinction coefficients and simultaneous equations for assaying chlorophylls a and b extracted with four different solvents: verification of the concentration of chlorophyll standards by atomic absorption spectroscopy. *Biochimica et Biophysica Acta (BBA) - Bioenergetics* 975:384–394. DOI: 10.1016/S0005-2728(89)80347-0.

Price JT, McLachlan RH, Jury CP, Toonen RJ, Grottoli AG. 2021. Isotopic approaches to estimating the contribution of heterotrophic sources to Hawaiian corals. *Limnology and Oceanography* 66:2393–2407. DOI: 10.1002/lno.11760.

Price KL, Peters EC. 2018. *Histological Techniques for Corals*. Kathy L. Price and Esther C. Peters.

Price J, Smith A, Dobson K, Grottoli AG. 2020. Airbrushed coral sample preparation for organic stable carbon and nitrogen isotope analysis. *Protocolsio*. DOI: 10.17504/protocols.io.bgi7juhn.

Pupier CA, Bednarz VN, Ferrier-Pagès C. 2018. Studies With Soft Corals – Recommendations on Sample Processing and Normalization Metrics. *Frontiers in Marine Science* 0. DOI: 10.3389/fmars.2018.00348.

Pupier CA, Grover R, Fine M, Rottier C, van de Water JAJM, Ferrier-Pagès C. 2021. Dissolved Nitrogen Acquisition in the Symbioses of Soft and Hard Corals With Symbiodiniaceae: A Key to Understanding Their Different Nutritional Strategies? *Frontiers in Microbiology* 12:657759. DOI: 10.3389/fmicb.2021.657759.

Putnam HM, Davidson JM, Gates RD. 2016. Ocean acidification influences host DNA methylation and phenotypic plasticity in environmentally susceptible corals. *Evolutionary Applications* 9:1165–1178. DOI: 10.1111/eva.12408.

Quinn TM, Crowley TJ, Taylor FW, Henin C, Joannot P, Join Y. 1998. A multicentury stable isotope record from a New Caledonia coral: Interannual and decadal sea surface temperature variability in the southwest Pacific since 1657 A.D. *Paleoceanography* 13:412–426. DOI: 10.1029/98PA00401.

Rahav O, Ben-Zion M, Achituv Y, Dubinsky Z. 1991. A photographic, computerized method for in situ growth measurements in reef-building cnidarians. *Coral Reefs* 9:204. DOI: 10.1007/BF00290422.

Rasband, W.S. 1997. *ImageJ*. Bethesda, Maryland, USA,: U. S. National Institutes of Health.

Reverter M, Tapissier-Bontemps N, Lecchini D, Banaigs B, Sasal P. 2018. Biological and Ecological Roles of External Fish Mucus: A Review. *Fishes* 3:41. DOI: 10.3390/fishes3040041.

Rinkevich B, Loya Y. 1979. The Reproduction of the Red Sea Coral Stylophora pistillata. I. Gonads and Planulae. *Marine Ecology Progress Series* 1:133–144.

Rodrigues LJ, Grottoli AG. 2006. Calcification rate and the stable carbon, oxygen, and nitrogen isotopes in the skeleton, host tissue, and zooxanthellae of bleached and recovering Hawaiian corals. *Geochimica et Cosmochimica Acta* 70:2781–2789. DOI: 10.1016/j.gca.2006.02.014.

Rodríguez-Casariego JA, Mercado-Molina AE, Garcia-Souto D, Ortiz-Rivera IM, Lopes C, Baums IB, Sabat AM, Eirin-Lopez JM. 2020. Genome-Wide DNA Methylation Analysis Reveals a Conserved Epigenetic Response to Seasonal Environmental Variation in the Staghorn Coral Acropora cervicornis. *Frontiers in Marine Science* 7:822. DOI: 10.3389/fmars.2020.560424.

Roy S, Llewellyn CA, Egeland ES, Johnsen G. 2011. *Phytoplankton Pigments: Characterization, Chemotaxonomy and Applications in Oceanography*. Cambridge University Press.

Rubin BER, Gibbons SM, Kennedy S, Hampton-Marcell J, Owens S, Gilbert JA. 2013. Investigating the Impact of Storage Conditions on Microbial Community Composition in Soil Samples. *PLOS ONE* 8:e70460. DOI: 10.1371/journal.pone.0070460.

Saenger C, Affek HP, Felis T, Thiagarajan N, Lough JM, Holcomb M. 2012. Carbonate clumped isotope variability in shallow water corals: Temperature dependence and growth-related vital effects. *Geochimica et Cosmochimica Acta* 99:224–242. DOI: 10.1016/j.gca.2012.09.035.

Schimak MP, Toenshoff ER, Bright M. 2012. Simultaneous 16S and 18S rRNA fluorescence in situ hybridization (FISH) on LR White sections demonstrated in Vestimentifera (Siboglinidae) tubeworms. *Acta Histochemica* 114:122–130. DOI: 10.1016/j.acthis.2011.03.008.

Shao W, Khin S, Kopp WC. 2012. Characterization of Effect of Repeated Freeze and Thaw Cycles on Stability of Genomic DNA Using Pulsed Field Gel Electrophoresis. *Biopreservation and Biobanking* 10:4–11. DOI: 10.1089/bio.2011.0016.

Shen GT, Boyle EA, Lea DW. 1987. Cadmium in corals as a tracer of historical upwelling and industrial fallout. *Nature* 328:794–796. DOI: 10.1038/328794a0.

Shinn EA. 1966. Coral growth-rate, an environmental indicator. *Journal of Paleontology* 40. DOI: 10.2110/palo.

Siebeck UE, Marshall NJ, Klüter A, Hoegh-Guldberg O. 2006. Monitoring coral bleaching using a colour reference card. *Coral Reefs* 25:453–460. DOI: 10.1007/s00338-006-0123-8.

Smith S V. 1973. Carbon Dioxide Dynamics: a Record of Organic Carbon Production, Respiration, and Calcification in the Eniwetok Reef Flat Community. *Limnology and Oceanography* 18:106–120. DOI: 10.4319/lo.1973.18.1.0106.

Southerland HA, Lewitus AJ. 2004. Physiological responses of estuarine phytoplankton to ultraviolet light-induced fluoranthene toxicity. *Journal of Experimental Marine Biology and Ecology* 298:303–322. DOI: 10.1016/S0022-0981(03)00364-2.

Stimson JS. 1985. The Effect of Shading by the Table Coral Acropora Hyacinthus on Understory Corals. *Ecology* 66:40–53.

Stimson JS, Kinzie RA. 1991. The temporal pattern and rate of release of zooxanthellae from the reef coral Pocillopora damicornis (Linnaeus) under nitrogen-enrichment and control conditions. *Journal of Experimental Marine Biology and Ecology* 153:63–74. DOI: 10.1016/S0022-0981(05)80006-1.

Storz D, Gischler E. 2011. Coral extension rates in the NW Indian Ocean I: Reconstruction of 20th century SST variability and monsoon current strength. *Geo-Marine Letters* 31:141–154. DOI: 10.1007/s00367-010-0221-z.

Sumner LW, Amberg A, Barrett D, Beale MH, Beger R, Daykin CA, Fan TW-M, Fiehn O, Goodacre R, Griffin JL, Hankemeier T, Hardy N, Harnly J, Higashi R, Kopka J, Lane AN, Lindon JC, Marriott P, Nicholls AW, Reily MD, Thaden JJ, Viant MR. 2007. Proposed minimum reporting standards for chemical analysis. *Metabolomics* 3:211–221. DOI: 10.1007/s11306-007-0082-2.

Swart PK, Greer L, Rosenheim BE, Moses CS, Waite AJ, Winter A, Dodge RE, Helmle K. 2010. The 13C Suess effect in scleractinian corals mirror changes in the anthropogenic CO2 inventory of the surface oceans. *Geophysical Research Letters* 37. DOI: 10.1029/2009GL041397.

Tambutté E, Allemand D, Bourge I, Gattuso JP, Jaubert J. 1995. An improved 45Ca protocol for investigating physiological mechanisms in coral calcification. *Marine Biology* 122:453–459. DOI: 10.1007/BF00350879.

Tambutté E, Allemand D, Zoccola D, Meibom A, Lotto S, Caminiti N, Tambutté S. 2007. Observations of the tissue-skeleton interface in the scleractinian coral Stylophora pistillata. *Coral Reefs* 26:517–529. DOI: 10.1007/s00338-007-0263-5.

Tanaka Y, Grottoli AG, Matsui Y, Suzuki A, Sakai K. 2015. Partitioning of nitrogen sources to algal endosymbionts of corals with long-term 15N-labelling and a mixing model. *Ecological Modelling* 309–310:163–169. DOI: 10.1016/j.ecolmodel.2015.04.017.

Tanzil JTI, Brown BE, Dunne RP, Lee JN, Kaandorp JA, Todd PA. 2013. Regional decline in growth rates of massive Porites corals in Southeast Asia. *Global Change Biology* 19:3011–3023. DOI: 10.1111/gcb.12279.

Teai T, Drollet JH, Bianchini J-P, Cambon A, Martin PMV. 1998. Occurrence of ultraviolet radiation-absorbing mycosporine-like amino acids in coral mucus and whole corals of French Polynesia. *Marine and Freshwater Research* 49:127–132. DOI: 10.1071/mf97051.

Torda G, Donelson JM, Aranda M, Barshis DJ, Bay L, Berumen ML, Bourne DG, Cantin N, Foret S, Matz M, Miller DJ, Moya A, Putnam HM, Ravasi T, van Oppen MJH, Thurber RV, Vidal-Dupiol J, Voolstra CR, Watson S-A, Whitelaw E, Willis BL, Munday PL. 2017. Rapid adaptive responses to climate change in corals. *Nature Climate Change* 7:627–636. DOI: 10.1038/nclimate3374.

Vago R, Gill E, Collingwood JC. 1997. Laser measurements of coral growth. *Nature* 386:30–31.

Voolstra CR, Buitrago-López C, Perna G, Cárdenas A, Hume BCC, Rädecker N, Barshis DJ. 2020. Standardized short-term acute heat stress assays resolve historical differences in coral thermotolerance across microhabitat reef sites. *Global Change Biology* 26:4328–4343. DOI: 10.1111/gcb.15148.

Wakai S, Shibuki Y, Yokozawa K, Nakamura S, Adegawa Y, Yoshida A, Tsuta K, Furuta K. 2014. Recycling and Long-Term Storage of Fluorescence In Situ Hybridization Slides. *American Journal of Clinical Pathology* 141:374–380. DOI: 10.1309/AJCPYX1UTI7LDAUY.

Wang XT, Cohen AL, Luu V, Ren H, Su Z, Haug GH, Sigman DM. 2018. Natural forcing of the North Atlantic nitrogen cycle in the Anthropocene. *Proceedings of the National Academy of Sciences* 115:10606–10611. DOI: 10.1073/pnas.1801049115.

Warner ME, Berry-Lowe S. 2006. Differential xanthophyll cycling and photochemical activity in symbiotic dinoflagellates in multiple locations of three species of Caribbean coral. *Journal of Experimental Marine Biology and Ecology* 339:86–95. DOI: 10.1016/j.jembe.2006.07.011.

Wellington GM, Dunbar RB, Merlen G. 1996. Calibration of stable oxygen isotope signatures in Galápagos corals. *Paleoceanography* 11:467–480. DOI: 10.1029/96PA01023.

Williams A, Chiles EN, Conetta D, Pathmanathan JS, Cleves PA, Putnam HM, Su X, Bhattacharya D. Metabolomic shifts associated with heat stress in coral holobionts. *Science Advances* 7:eabd4210. DOI: 10.1126/sciadv.abd4210.

Winters G, Holzman R, Blekhman A, Beer S, Loya Y. 2009. Photographic assessment of coral chlorophyll contents: Implications for ecophysiological studies and coral monitoring. *Journal of Experimental Marine Biology and Ecology* 380:25–35. DOI: 10.1016/j.jembe.2009.09.004.

Work T, Meteyer C. 2014. To Understand Coral Disease, Look at Coral Cells. *EcoHealth* 11:610–618. DOI: 10.1007/s10393-014-0931-1.

Wright RM, Correa AMS, Quigley LA, Santiago-Vázquez LZ, Shamberger KEF, Davies SW. 2019. Gene Expression of Endangered Coral (Orbicella spp.) in Flower Garden Banks National Marine Sanctuary After Hurricane Harvey. *Frontiers in Marine Science* 6:672. DOI: 10.3389/fmars.2019.00672.

Zhou J, Fan T-Y, Beardall J, Gao K. 2016. Incident Ultraviolet Irradiances Influence Physiology, Development and Settlement of Larva in the Coral Pocillopora damicornis. *Photochemistry and Photobiology* 92:293–300. DOI: 10.1111/php.12567.
